# An evidence-based critical review of the mind-brain identity theory

**DOI:** 10.3389/fpsyg.2023.1150605

**Published:** 2023-10-27

**Authors:** Marco Masi

**Affiliations:** Independent Researcher, Knetzgau, Germany

**Keywords:** philosophy of mind, mind–body problem, psychology, neuroscience, material monism, physicalism, dualism

## Abstract

In the philosophy of mind, neuroscience, and psychology, the causal relationship between phenomenal consciousness, mentation, and brain states has always been a matter of debate. On the one hand, material monism posits consciousness and mind as pure brain epiphenomena. One of its most stringent lines of reasoning relies on a ‘loss-of-function lesion premise,’ according to which, since brain lesions and neurochemical modifications lead to cognitive impairment and/or altered states of consciousness, there is no reason to doubt the mind-brain identity. On the other hand, dualism or idealism (in one form or another) regard consciousness and mind as something other than the sole product of cerebral activity pointing at the ineffable, undefinable, and seemingly unphysical nature of our subjective qualitative experiences and its related mental dimension. Here, several neuroscientific findings are reviewed that question the idea that posits phenomenal experience as an emergent property of brain activity, and argue that the premise of material monism is based on a logical correlation-causation fallacy. While these (mostly ignored) findings, if considered separately from each other, could, in principle, be recast into a physicalist paradigm, once viewed from an integral perspective, they substantiate equally well an ontology that posits mind and consciousness as a primal phenomenon.

## 1. Introduction

Since the times of René Descartes in the 17th century, the mind–body problem has been one of the central debates in the philosophy of mind, psychology, and neuroscience. The conventional Cartesian dualism is no longer considered tenable but other forms of dualism, or theoretical frameworks of philosophical idealism, or more generally, non-physicalist ontologies, state that mind and consciousness cannot be explained as a mere result of neural processes.

Dualism is opposed by an identity theory, which, instead, considers mind processes as identical to brain processes, and consciousness as nothing other than an emergent epiphenomenon arising from the collective interaction of the neuronal activity. Sentience, with all its subjective dimensions of experiences, feelings, and thoughts, is a physical process determined only by the laws of physics. Qualia–the subjective, phenomenal, and mental experiences we can access only introspectively, such as the perception of color, or that of pain and pleasure–are physical brain states, while any speculation concerning an immaterial mind or consciousness is considered an unnecessary hypothesis.

Dualists and monists have different schools of thought but, despite the variety of opinions, it is fair to say that most scientists and philosophers consider themselves to be material monists. For example, according to a survey (Bourget and Chalmers, 2020) 51.9% of philosophers declare themselves ‘physicalists’ vs. 32.1% as non-physicalists, and 15.9% as ‘other’. On the other hand, exceptional human experiences occur frequently in both the general population and in scientists and engineers (Wahbeh et al., 2018).

However, there is a growing awareness that a mere functional investigation will not answer questions of a more philosophical nature. The belief that the progress of modern neurosciences would soon shed light on David Chalmer’s notorious ‘hard problem of consciousness’ (Chalmers, 1995) has turned out to be too optimistic. This is because, unlike other physical processes, in which both causes and effects can be observed from a third-person perspective, in consciousness studies, one is confronted with a cause–the brain activity–that one can still analyze from a third-person perspective that, however, apparently produces an effect we call ‘conscious experience,’ or just ‘sentience,’ which can be apprehended only from a first-person perspective. This ‘perspectival asymmetry’ makes consciousness in its subjective and experiential dimension stand out as a phenomenon alien to any attempt at conceptual causal and ontological scientific reduction. Inside a naturalistic framework, the origin and ontology of the phenomenal subjective conscious experience remain unclear.

While most arguments were based on a physicalist line of reasoning (for a review, see (Seth and Bayne, 2022)), and also other post-materialistic models of consciousness that are not exclusively based on brain activity exist (for a review and discussion see (Wahbeh et al., 2022)), here it is shown that there are also strictly neuroscientific facts that have not received sufficient appreciation and that give us good reasons to look upon the physicalist assumptions with a more critical eye. Non-neurocentric paradigms of consciousness that posit mind and consciousness as a fundamental primitive, rather than matter, remain a viable option. No particular dualistic, panpsychist, Eastern philosophical, or metaphysical scheme is favored. Rather, a variety of findings, especially when seen jointly and in their relationship to each other, could suggest other possible ways of interpreting the neuroscientific findings, and that this might even have more explanatory power in terms of an underlying post-material ontology.

A preliminary note of conceptual and terminological clarity is necessary. In psychology, or the philosophy of mind, and neurological sciences, the words ‘consciousness,’ ‘mind’, and ‘self-awareness’ are defined and used with different significances, sometimes with overlapping or conflating semantics. In fact, for historical reasons, the mind-brain identity theory used the terms ‘mind’ and ‘consciousness’ somewhat interchangeably (Smart, 2022). Here, however, ‘consciousness’ will relate to phenomenal consciousness–that is, Nagel’s famous ‘what-it-is-like’ states (Nagel, 1974) underlying our subjective qualitative experiences, ‘qualia,’ that what makes us sentient of perceptions, feelings, sensations, pleasures or pains, and self-aware as a unified subject. Phenomenal consciousness is not to be confused with ‘mind’ which, at least in the present context, relates to the cognitive functions of thought, memory, intelligence, ideas, concepts, and meanings. The two are to be kept distinct in the sense that the mind’s thoughts come and go, while the conscious experiencing subject is permanent. I deem this distinction necessary because the question relating to the physicality of the spectrum of all our psychological dimensions, as we are going to see later, may not have a unique answer. For example, one can argue for the unphysical nature of phenomenal consciousness but maintain that memory is in the brain, or that low-level cognition (e.g., sensory perception modalities) are neuronal epiphenomena, while other high-level functions (decision-making, agency, reasoning, and planning) are not.

Having made this distinction, in the following, I will first examine more closely the logical framework that sustains a mechanistic conception by pointing out some conventional neurological causation-correlation fallacies.

Let us first question some basic assumptions. Does the physical change of a brain state leading to cognitive impairment or altered states of consciousness provide a necessary and sufficient logical proof that mind and consciousness are an emergent cerebral phenomenon?

After all, it is undeniable that there is a direct relation between the physical state of our brains and our subjective experiences (e.g., Aguinaga et al., 2018), (Vollenweider and Preller, 2020), (Davis et al., 2008). Dopamine is a neurotransmitter molecule that enables biochemical transmission among neurons and that is responsible for the effects of a drug like cocaine. We know that psychedelic drugs can lead to intense subjective effects. It is a well-known fact that brain damage can lead to severe cognitive impairments. If Broca’s area, a left cerebral hemisphere area, is lesioned, one loses the ability to speak (interestingly, though, not the ability to comprehend language). Someone being anesthetized using anesthetic drugs (seemingly) ‘loses’ consciousness. And nowadays, we have a number of sophisticated brain scan technologies making it clear, beyond any reasonable doubt, that for every conscious experience, there exists a neural correlate in our brains.

Thus, apparently, a neuroscience that is based on brain chemistry and loss-of-function lesion studies leaves no place for any form of non-material monistic approach. Mental states and conscious self-awareness seem to emerge from matter; there is no distinction. Our personalities, identities, moods, and states of consciousness seem to depend on the biophysical state of our brains.

And yet, few further critical thoughts should make it clear that such a correlation is not a sufficiency criterion. One must secure one’s theoretical framework from a possible logical fallacy believing that correlation implies causation. The fact that two events are always coincidental or always happen shortly, one after the other, does not imply that the first event caused the second event to happen. If event B always follows event A, we are not entitled to conclude that A is the cause of B. These sorts of logical fallacies are known as ‘post-hoc fallacies’.

Nevertheless, the necessity and sufficiency that the explanation of our qualitative experiential dimension is to be chiefly found in neural circuits remains a rarely questioned belief [with few exceptions, e.g., in the field of behavioral processes (Gomez-Marin, 2017)]. There is a general tendency to believe that causal mechanistic explanations based on neural lower-level properties are better than higher-level behavioral accounts. For example, Krakauer et al. pointed out that neuroscientists (and, I would add, too many psychologists and most analytical philosophers of mind) frequently use language to hide more than to reveal, by assuming that a neural causal efficacy equals understanding–that is, charging it with an explanatory power it does not have. The result that “neural activity X is necessary and sufficient for behavior Y to occur” allows a causal claim often added by a further explanatory sentence that rearticulates the same causal result employing ‘filter verbs’ (such as “produces, “generates,” “enables,” etc.) and that, however, masks the faulty logic to cause a metaphysical position to pass as empirical data (Krakauer et al., 2017).

But, what are the alternatives to the mind–body identification that could be in line with the above correlation between mental states and physical neural correlates of consciousness?

In fact, the metaphor most idealists prefer is the ‘filter theory of consciousness,’ which dates back to an original idea of William James, who stated: *“My thesis is now this: that, when we think of the law that thought is a function of the brain, we are not required to think of productive function only; **we are entitled also to consider permissive or transmissive function**. And the ordinary psycho-physiologist leaves this out of his account*” (emphasis in the original text) (James, 1898).

James thought of the brain and thought in the frame of a ‘bidirectional transducer theory’ using the analogy of the prism separating white light into respective colored beams. If a broken prism fails in its function to ‘reveal’ the colored light beams, this should not lure us into the logical correlation-causation fallacy that the prism ‘produces’ colored light. The material and structural modification of the optical medium modifies the refractive gradient that ‘transduces’ light with a different chromatic dispersion but does not ‘create’ it. A prism is just an object with a transmissive function; it does not ‘generate’ anything.

Aldous Huxley expressed a similar idea and proposed that the brain is a ‘reducing valve’ of what he called a ‘Mind at large,’ a universal or cosmic Mind comprising all of reality with all ideas and all thoughts. According to Huxley, our mind filters reality under normal conditions because, otherwise, we would be overwhelmed by the knowledge of this universal Mind. Psychedelic drugs can remove the filter and bring us into contact with the Mind at large, leading to the experiences that several mystics describe. In his words: *“To make survival possible biologically, Mind at large has to be funneled through the reducing valve of the brain and nervous system”* (Huxley, 1954). For Huxley, the brain was a material ‘connecting device,’ an ‘interface’ or ‘relay station.’ In this view, human mind is a localization of a universe-wide Mind projected into our brains. The brain filters and suppresses this universal Mind but does not ‘produce’ it.

An understanding of the mind-brain relationship reminiscent of Eastern philosophies, and that maintains similar views, is neatly summarized by the Indian mystic and poet Sri Aurobindo: *“Our physical organism no more causes or explains thought and consciousness than the construction of an engine causes or explains the motive-power of steam or electricity. The force is anterior, not the physical instrument”* (Aurobindo, 1919).

From these perspectives, mind uses the brain as an instrument, as an interface of expression. Mind and consciousness are constrained and interdependent from the brain but aren’t generated by the instrument itself.

Notice that this standpoint is not entirely alien to our ordinary understanding of how a digital computer works. Knowing everything about its hardware, and recreating its exact physical structure in every detail, would not lead us to a machine that makes anything meaningful or useful. Software–that is, a running code written by an intelligent external agent–is needed. Here, also, a computer is only an instrument, a means of expression for a cognitive entity, not its origin or source. In fact, studying a microprocessor with the same criteria employed by modern neuroscience, trying to reverse-engineer its functions by analyzing local field potentials, or selectively lesioning its units by correlating this with its behavior, would turn out to be a quite difficult task: We would still have a long way to go to explain how it works and figure out the whole running code, which is the real ‘agent’ causing the behavior of the machine (Jonas and Kording, 2017).

Thus, neural correlates of consciousness, or loss-of-function lesion-based studies, do not constitute a sufficient logical foundation for a mind-brain identity theory. We have the right to maintain the contrary hypothesis: Consciousness, mental states, and emotional states are more or less ‘funneled through’ depending on the physical state of a brain. The brain could equally well be seen as a physical substrate *through which* these conscious states manifest without leading to any inconsistency with current scientific knowledge. How current neuroscience not only fails to falsify this hypothesis but maybe even suggestive of this claim, is the purpose of the next section, with a review of old and new neuroscientific findings that are asking for clarification if one wants to save the mind-consciousness-brain identity theory. Another part will review the evidence for the neural correlates of memory. A brief section will focus on the emergent fields in the study of plant and cellular ‘basal cognition.’ A discussion and concluding remarks will follow.

## 2. From (lack of) evidence to interpretation

### 2.1. The search for the ‘seat of consciousness’

Crick and Koch once postulated that the claustrum, a sheet-like neuronal structure hidden beneath the inner surface of the neocortex, might give rise to “integrated conscious percepts”–that is, act like the “seat of consciousness” (Crick and Koch, 2005). Modern neuroscience, however, indicates that the claustrum behaves more like a neuronal information router than an organ responsible for a specific function (Madden et al., 2022). To date, there is no evidence, not even indirect or circumstantial, of a single brain region, area, organ, anatomical feature, or modern Cartesian pineal gland that takes charge of this mysterious job of ‘producing’ or ‘generating’ consciousness. Most of the brain is busy processing sensory inputs, motor tasks, and automatic and sub- or unconscious physiological regulations (such as the heartbeat, breathing, the control of blood pressure and temperature, motor control, etc.) that do not lead to qualitative experiences. Neural activity alone cannot be a sufficient condition to lead to phenomenal consciousness. The vast majority of brain activity is unconscious–that is, non-conscious cognitive processes (e.g., mnemonic, perceptual, mental or linguistic tasks) and physiological processes (e.g., cardiac, hormonal, thermal regulation, etc.) taking place outside of our conscious awareness. This raises the question: What distinguishes a neural process that leads to a conscious experience from that which does not?

For example, the cerebellum is almost exclusively dedicated to motor control functions, and its impairment leads to equilibrium and movement disorders. However, it does not affect one’s state of consciousness. Its role in ‘generating’ experience seems to be marginal, if any. There are also rare cases of people who live without a cerebellum (‘cerebellar agenesis’) and have only mild or moderate motor deficits or other types of disorders (Feng et al., 2015). This is a fact that seemingly confirms the brain’s proverbial neuro-plasticity, which we will see next through other extraordinary examples.

**Figure 1:**
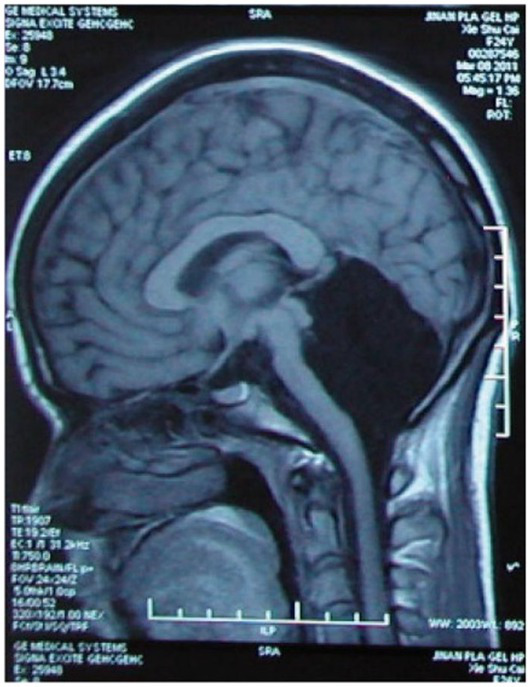
Case of cerebellar agenesis: Living (and walking) without the cerebellum. Credit: Feng et al. (2015). Reproduced with permission of Oxford University Press.

It may be worth recalling that the neuronal architecture in our bodies is not confined to the brain–that is, it goes far beyond our heads, through the brain stem, and down through the spinal cord. The central nervous system is made up of the brain and the spinal cord. The latter is responsible for the transmission of nerve signals from and to the motor cortex; as is well known, injury to it can result in paralysis. But, again, no cognitive deficit or state of consciousness is altered by impairments of the spinal cord. This leaves only one option: If there is a ‘seat of consciousness,’ it must be identified somewhere in the cerebral cortex or subcortical areas of the brain (Figure 1).

Another interesting example of how the correlation-causation fallacy conditions scientific and popular understanding of the mind–body problem can be illustrated by an interesting experimental finding that showed how stimulation of the thalamus arouses macaques from stable anesthesia (Redinbaugh et al., 2020). The awake, sleeping, and anesthetized states could be aroused with the stimulation of the central lateral thalamus. The straightforward conclusion seemed clear. The ultimate origin and switch ‘modulating’ consciousness was discovered. If your consciousness ‘depends’ on the state of your thalamus, which is ‘switched’ on and off with the touch of a button, then the thalamus must be the ‘seat of consciousness.’ Is this an unavoidable conclusion?

First of all, observing from a third-person perspective the absence of an external physiological signature as evidence for a lack of internal first-person sentience is yet another correlation-causation fallacy that has too frequently led to unwarranted conclusions. For example, that anesthesia induces an unconscious state with the patient having no subjective experience is far from obvious. We simply do not know if it really induces a completely unconscious state or a conscious but non-metacognitive no-report state that makes one unable to recall past experiences once one is back in the waking state. The former assumption is, unfortunately, taken in most cases as the standard scientific approach. Whereas, indications suggest that anesthetic-induced unresponsiveness does not induce complete disconnectedness (Radek et al., 2018; Turku, 2018). Interestingly in this regard is also the so-called twilight anesthesia, an anesthetic technique that sedates patients only mildly and induces amnesia but no loss of consciousness (Scheinin et al., 2020). During this ‘twilight state,’ patients are responsive and can be asked to perform some tasks that they will not be able to recollect after the surgery. This case alone shows that the inability to recall events during sedation is no proof of unconsciousness.

Moreover, there is now a non-negligible amount of scientific literature, presenting empiric evidence on parasomnia (sleepwalking), hypnosis, non-REM sleep, and subjects in a vegetative state, that some form of conscious awareness is also present in all these non-responsive states of consciousness (e.g., Owen and Coleman, 2006; Oudiette et al., 2009; Cruse et al., 2011; Siclari et al., 2018; Mackenzie, 2019). Arguing and extrapolating from the lack of superficial physical cues and mnemonic retention to a verdict that declares someone to be ‘unconscious’–that is, as having no subjective phenomenal experience–is, at least from the philosophical perspective, again betraying a logical correlation-causation fallacy.

But even if we assume that there is no internal experience when we are anesthetized, the relevant question remains: Do these sorts of experimental findings confirm that the thalamus is the ‘seat of consciousness’? Is it a sort of modern replacement for Descartes’ pineal gland in its mechanistic-material monist version?

The thalamus is responsible for sensory information processing. It is known that its main job is to function as a relay and feedback station between sensory brain areas and the cerebral cortex. For example, it functions as a hub between the optical nerves that transport the visual information coming from our retinas to the visual cortex. Even if one remained conscious by turning down the functionality of the thalamus, one would no longer see anything because the neural pathways between the retina and the visual cortex are interrupted. From that, however, nobody would conclude that the thalamus is the seat of the visual experience for which the visual cortex is responsible, as we know that it is a ‘hub,’ a ‘transducer’ or a ‘filter.’ From this perspective, the thalamus’ function is to ‘integrate’ the information flow of the several brain areas; if this is disrupted, it leads to a ‘loss’ of consciousness.

Thus, these findings do not tell us much about the generation of conscious experience. However, if there is not one single ‘seat of consciousness,’ could it be that the combination and activity of some or all of the different brain areas do ‘produce’ the subjective experience? Considerable attention in this direction has been focused on theories such as the ‘Integrated Information Theory’ (IIT) (Oizumi et al., 2014; Tononi, 2015) and the ‘Global Workspace Theory’ (GWT) (Baars, 1988), according to which the amount and integration of information and the momentarily active and accessible memory determine the level of consciousness leading to a conscious entity. A process of integrating the information and the memory coming from all the brain areas may be the efficient cause of our experiential richness. In fact, we have sufficient evidence that compels us to abandon this simplistic view of a compartmentalized brain, with modern neuroscience thinking more in terms of network science, in which several brain regions are highly interconnected and interdependent. No brain region does only one thing, and no neurons supposedly have only one function. Most neurons have several functions, not a single purpose. It turns out that whenever we hear a sound, have a visual experience, have feelings or emotions, or perform a motoric task, the whole brain is involved. Even such an apparently highly specialized brain region as the primary visual cortex carries out information processes related to hearing, touch, and movement (Merabet et al., 2008; Liang et al., 2013). The reason why we nevertheless tend to associate specific brain regions with specific cognitive, sensorial, or motoric functions is that brain scans show only a temporal snapshot of the brain’s most intense activity. We are seeing only a few ‘tips of the iceberg’ and missing the overall activity in the noise. When studies are conducted using less noisy but much more expensive and complicated detection methods, most of the brain’s activity becomes visible (Gonzalez-Castillo et al., 2012). Therefore, it would seem plausible that if consciousness arises from the activity of a complex aggregation of neurons, at least some brain areas must work together in a unified whole via thalamic activity.

However, how far these conjectures align with reality is questionable.

**Figure 2:**
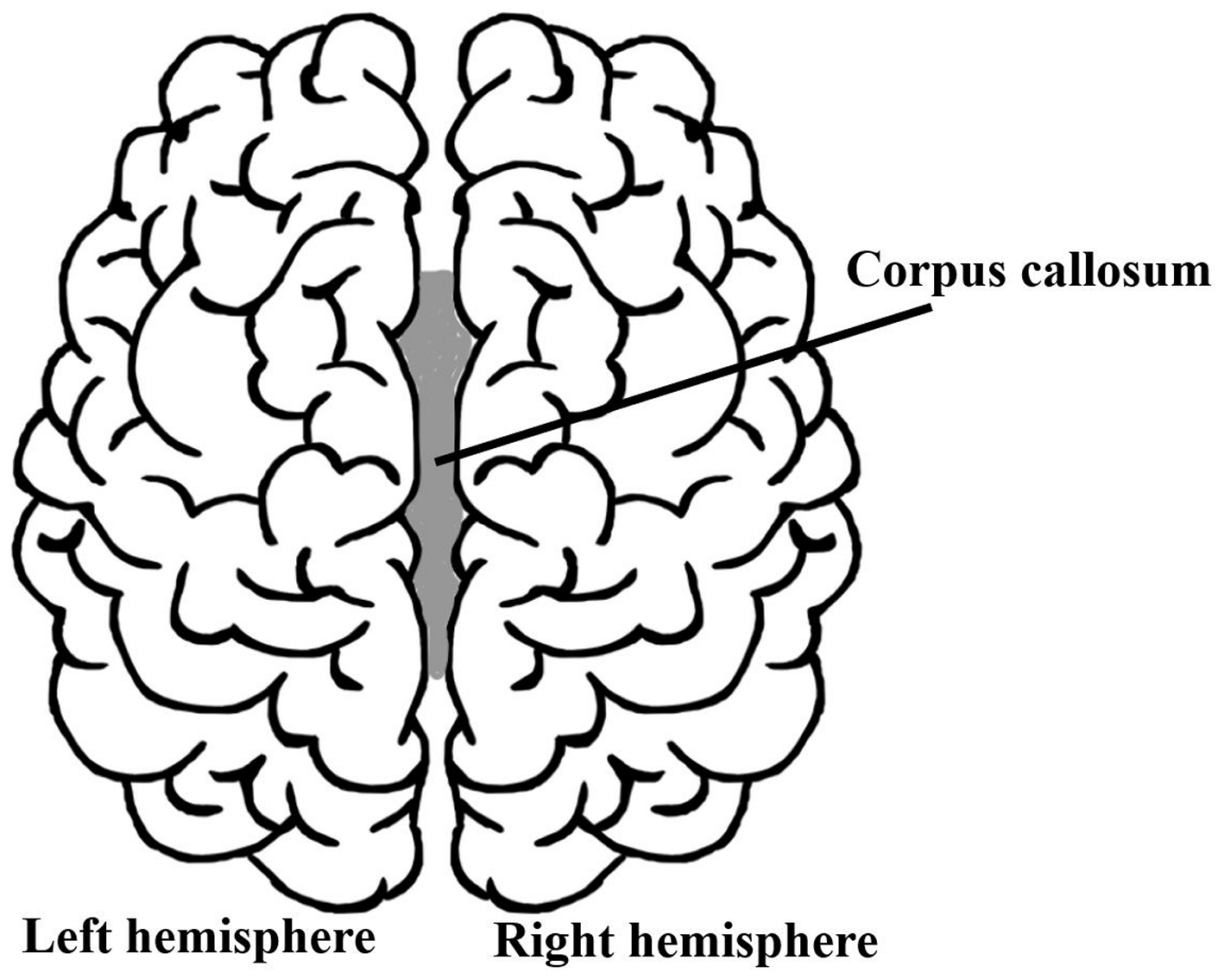
Does brain-splitting cause ‘self-splitting’?

Because a natural question could be that of asking if and how a subjective feeling of selfhood changes if someone were to split your brain into two parts? Would you feel somewhat less conscious and less ‘yourself’? As is well-known, this is a very real surgical procedure performed since the 1940s: the corpus callosotomy (although used only rarely nowadays). It is performed to treat the worst cases of epilepsy (patients having up to 30 seizures a day) that did not respond to medical treatment. In this procedure, the corpus callosum, the nerve tract connecting the left and right brain hemispheres, is severed (in part or, in some cases, entirely), thereby avoiding the spread of epileptic activity between the two halves of the brain (Figure 2). Its natural function is to ensure communication between the two cerebral cortexes of the two hemispheres to integrate and coordinate motor, sensory, and cognitive functions, such as moving left and right limbs, the visual integration of the left and right sight, etc. Because most of the brain’s activity is distributed throughout both hemispheres, with no indication of one or the other part being responsible for generating our sense of ‘self,’ one must wonder how the patients who have gone through such an acute surgical intervention feel. Do their split brains ‘generate’ a dual consciousness and split personality?

Disagreement exists about whether in these patients a subject unity is present or if they display any signs of multiple first-person perspectives (De Haan et al., 2020). They deny being a different person from what they were before surgery, and close relatives who knew the split-brain patients before and after surgery do not notice any personality change (Bogen et al., 1965; Sperry, 1968, 1984; Pinto et al., 2017),

Of course, there can be more or less severe drawbacks. In some cases, the so-called ‘alien-hand syndrome’ can take over, in which one hand appears to have a mind of its own. This occasionally happens when the two hemispheres’ representations of reality come into conflict and one wants to override the other. In these instances, decision-making and volition between the two hemispheres clash. An example is the patient‘s struggle to overcome an antagonistic behavior, such as knowing what cloth they want to wear, while one of their hands takes control and reaches out for another cloth they do not want at all. However, this should not be confused with two personalities competing against each other (as in the case of dissociative identity disorders), as split-brain patients identify with only one body and perceive their disobedient limb as being subjected to annoying motoric misbehavior; they do not report any sensation of some other internal personality taking control. The brain–or, more precisely, our two brains–tell us two different ‘stories.’ Split-brain patients seem to identify with one of the stories–that is, consciously access one of its interpretations–and keep the other in a subconscious or subliminal awareness, what the American cognitive neuroscientist Michael Gazzaniga used to call the ‘left-brain interpreter.’

Recent investigations also question the canonical textbook findings (Pinto et al., 2017, 2020). While it is confirmed that a corpus callosotomy splits the visual perception of the environment in two, several patients can nevertheless see them both and report it to the outside world–that is, they can access their language centers. Moreover, there is no evidence for memory loss (Forsdyke, 2015).

In my view, confusion surrounding split-brain psychology arises only if we conflate the ‘unity of mind’ with a ‘unity of consciousness’ and sense of selfhood. If we do not confuse mental states as being the origin or efficient cause of consciousness, then any apparent paradox dissipates. Split-brainers may have two (eventually even conflicting) hemispheric and motor-sensory mental states (something not entirely unusual in healthy subjects) but even if one argues and provides evidence for a ‘two-minds’ model, that would not imply a split sense of identity or self-awareness. One can consciously and subliminally be aware of a plurality of experiences, yet retain the experience of singularity. There can be several experiences and representations generated in a brain, with or without a representational unity, which, nevertheless, belongs to and is experienced by one subject [for a more detailed analysis of this point see (De Haan et al., 2021)]. A ‘split subjective identity’ resulting from split-brain in the sense of a symptomatology similar to what we know from dissociative identity disorder characterized by the disruption of identity in two distinct personalities, differing not just in sensory-motor functioning or depersonalization disorders, but also each with two psychological behaviors, characters, affects, social preferences, and experienced as alternating ‘possessions’ with cognitive discontinuities and different memories of autobiographical information, as observed by others and reported by the (alternating) subjects themselves, is not observed.

So, if our subjective and conscious experience is generated by the integrated activity of the whole brain, why does not such a radical bisection lead to any modification of our state of awareness? Given the severing of the corpus callosum of a brain, one would expect a loss or at least a diminishing of conscious awareness because there would be a loss of working memory and information integration. However, nothing like this happens. The ‘unity of consciousness’ remains unaffected and, thereby, unexplained.

To save the paradigm, those who endorse the view that in such brain condition consciousness can no longer be ‘integrated’, point out that in not all documented cases was a complete transection of the corpus callosum performed. The truth, however, is that in several cases, the complete sectioning was performed and even confirmed by MRI imaging or radiological means (Gazzaniga, 1985).

Yet, one may still point out that a complete transection still leaves some residual subcortical structures intact, which allows for some communication between the two hemispheres, potentially maintaining the ‘self’ of the patients.

**Figure 3:**
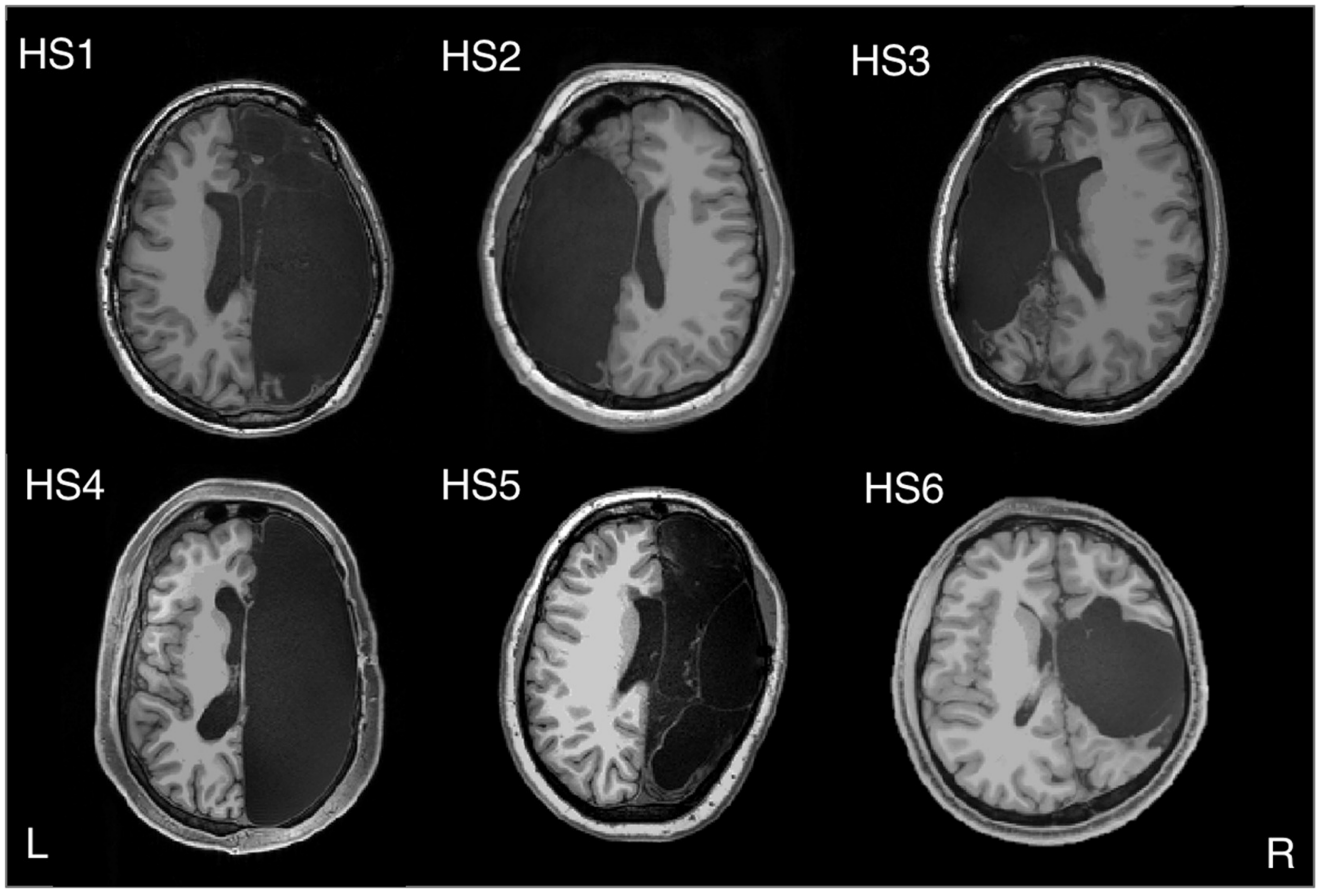
Hemispherectomy Brain Anatomy - Six adults with left (HS2 and HS3) or right (HS1, HS4, HS5, and HS6) hemispherectomy. Credit: Kliemann et al. (2019). Reproduced under the terms of CC BY NC ND.

To further substantiate the contrary hypothesis, one could mention cases in which there is no second hemisphere to communicate with in the first place. To treat epilepsy, the most extreme surgical intervention is to remove an entire brain hemisphere, that is, by hemispherectomy. Usually, this is done only in childhood because, supposedly, young brains can rewire themselves much more efficiently than older ones. Figure 3 shows the fMRI in a sample of six rare high-functioning patients after partial or complete surgical removal of one cerebral hemisphere.

**Figure 4:**
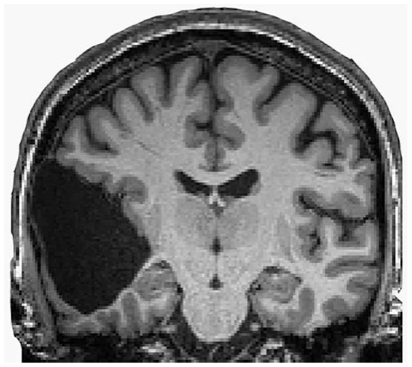
Speaking without the brain’s language area. Credit: Tuckute et al. (2022). Copyright 2022, reproduced with permission from Elsevier.

Interestingly, Nature seems to take the left/right distinction and early plasticity hypothesis not so seriously. That the left–right brain task distribution is not an inescapable neurological dogma is testified to by people born with only one hemisphere. For example, while in healthy subjects the left visual field is represented in the right hemisphere and vice versa, someone born with only one hemisphere can develop maps of both visual fields in it (Muckli et al., 2009). Hemispherectomy on adults older than 18 years turns out to be just as safe and effective as in early childhood (McGovern et al., 2019). Even in the case of a left hemispherectomy, Broca’s language area–which in normal conditions is in the left hemisphere–can be recovered in the right part of the brain (Vargha-Khadem et al., 1997). Further evidence reports of subjects in whom the frontal lobe was missing from childhood without any measurable linguistic impairments, as shown by the case of a woman who grew up without her left temporal lobe but speaks in English and Russian (Tuckute et al., 2022; Figure 4). This does not mean that persons missing a hemisphere do not suffer consequences–there is suboptimal word and face recognition (Granovetter et al., 2022) but whether it plays a role in the unity of consciousness remains to be seen.

A possible explanation is that because these patients already had severe seizures originating in one of the hemispheres, the functional rewiring on the other hemisphere began before the surgery. The findings tend to disconfirm this easy way out. Though interconnectivity inside the brain networks increases, interconnectivity between brain regions with the same function after hemispherectomy does not differ from that of two hemispheric control subjects (Kliemann et al., 2019). That plasticity alone can explain this state of affairs is far from proven (more on this later).

However, it is, most patients become seizure-free, and their cognition is relatively unchanged after surgery (some motoric and cognitive functions decrease but others improve). Overall, these patients appear to be ‘normal.’ Cognitive measures typically changed little between surgery and follow-up (Pulsifer et al., 2004), and in everyday life, one could not tell the difference between humans having a whole brain or only half of one. And, most notably, the subjects report no ‘half-self,’ ‘half-awareness,’ or ‘half-consciousness.’

If the mind-brain identity theory is correct, and consciousness emerges as an integration of functional centers, with no particular ‘seat of consciousness,’ then only one brain hemisphere must be sufficient to accomplish the task.

But instances are found in which both hemispheres are severely damaged and there is not much left to integrate. Worth a reminder is how, in 1980, the British pediatrician John Lorber reported that some adults cured of childhood hydrocephaly had no more than 5% volume of brain tissue with a cerebral cortex as thin as 1 mm (Lewin, 1980). While some had cognitive and perceptual disorders and several developed epilepsy, others were surprisingly asymptomatic and even of above-average intelligence.

**Figure 5:**
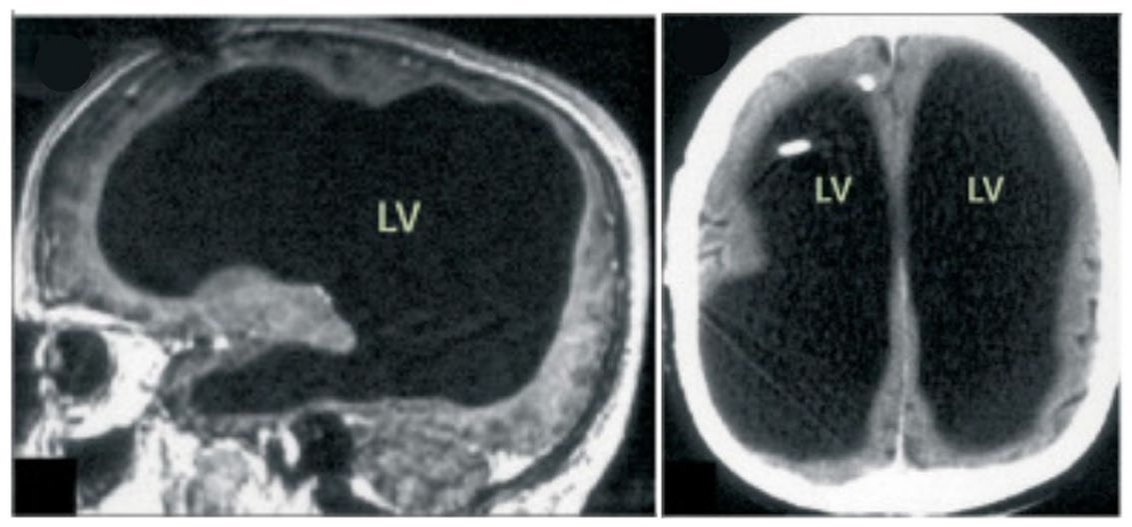
MRI image of a hydrocephalus brain. Credit: Feuillet et al. (2007). Copyright 2022, reproduced with permission from Elsevier.

Then, in 2007, in Marseille, France, a 44-year-old man complaining of weakness in his left leg submitted to an MRI brain scan (Feuillet et al., 2007). As Figure 5 shows, the skull was abnormally filled with cerebrospinal fluid, leaving only a thin sheet of actual brain tissue. As an infant, he’d had a shunt inserted into his head to drain the fluid but it was removed when he was 14. Evidently, the cerebrospinal fluid build-up did not stop and ended up reducing the brain’s size to 50–75% compared to its normal volume. Though he had a below-average IQ (75/100), this man had a job, a family, and a normal life.

Another example that should raise doubts is the cases of children in a developmental vegetative state–that is, what the American Academy of Neurology (as declared in its guideline report in 1995 and confirmed in 2018) officially considers as being a neurovegetative state in which there is *“no evidence of purposeful behavior suggesting awareness of self or environment”* (Giacino et al., 2018). In other words, a universal rule reduces them to unconscious children who cannot suffer because this supposedly requires a functioning cerebral cortex.

Nevertheless, only one case showing the contrary should be sufficient to disprove a universal rule. Four such cases were brought to light in 1999 by a group led by Shewmon et al. (1999). They studied the states of awareness in congenitally decorticate children–that is, the cases of four children who were almost completely lacking cortical tissue and were neurologically certified as being in a vegetative state. Yet, the loving care of their mothers (or of someone who adopted them and bonded with them via dedicated full-time caring) could gradually ‘awaken’ in them a conscious awareness. From an initially unresponsive state, they showed clear signs of having developed auditory perception and visual awareness (despite the total absence of the occipital lobe that, in normal conditions, hosts the visual areas). For example, they tracked faces and toys, looked at persons they recognized, could distinguish between their mothers or caretakers, listened to music for which they manifested preferences with their facial expressions, including smiling and crying, and, at least in one case, gave clear indications of self-recognition in a mirror. Shewmoon notes: *“Were they* [the decorticate children] *not humans studied by clinicians but rather animals studied by ethologists, no one would object to attributing to them ‘consciousness’ (or ability to ‘experience’ pain or suffering) based on their evident adaptive interaction with the environment.”*

These cases seem to contradict the prevailing theory, according to which the cerebral cortex generates consciousness.

**Figure 6:**
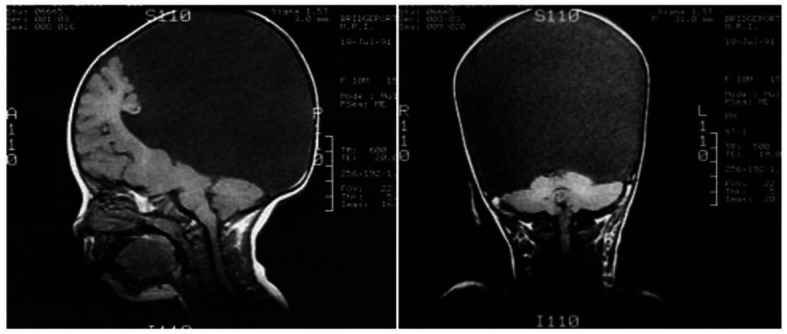
Congenitally decorticate children MRI brain scan (midline sagittal and posterior coronal plane). Credit: Shewmon et al. (1999). Reproduced with permission from Wiley.

One can still point out that the children were not completely decorticated, as some cortical tissue was still left. Figure 6 shows that a remnant of the frontal lobe is still present, possibly producing the conscious awareness. But that neural mechanisms of conscious function cannot be confined to the cerebral cortex alone is becoming much more plausible (Merker, 2007).

In fact, other speculations now retire to the last cerebral bastion for the seat of consciousness: the brainstem (Solms and Panksepp, 2012). Indeed, its stimulation can trigger intense emotions and feelings. But the question is: What property of a neural circuitry dedicated to the most physical and basal control of cardiac, respiratory, and homeostatic functions, containing mainly neurons for motor and sensory tasks, can also give rise to such an apparently immaterial and completely different and unrelated ‘function’ or ‘property’ as a conscious experience? We do not know. However, this is yet another fact telling us that we have the right, at least hypothetically, to assume that they do not and are equally allowed to study these facts in the light of a different paradigm than that of a mind-brain identity.

Overall, the cases mentioned above (except for those of the congenitally decorticate children) of people who have undergone corpus callosotomy or hemispherectomy, or people suffering from hydrocephalus, cerebellar agenesis, or several other types of brain damage, show how surprisingly intact their higher cognitive functions remain. One would expect that the first victims of such invasive neurological changes or surgical interventions would be the complex and high-demanding cognitive functions so characteristic of the mind, such as intellectual skills, abstract thinking, decision-making, reason, logically and willfully planning actions, and so on. Instead, it turns out that even if large brain masses are injured or absent, the cognitive skills of the subject remain substantially unaltered. Further empirical inquiry is needed to show if the same holds for the integrity of subjective experience and no altered states of consciousness or qualitative changes of sensory perception arise.

### 2.2. Further questions on the mind-brain relationship

These remarkable cases also confirm that brain size and the number of neurons in a brain do not (or, at least, do not necessarily) indicate one’s intelligence. Size matters for manipulative complexity, such as the more complex hand movements in primates, which humans can develop superbly (think of the hands of an expert musician playing piano; Heldstab et al., 2020). However, a direct correlation between brain size and mental skills is not that straightforward. We like to believe that our brain size makes us human but rarely do we question what one means by ‘size.’ The number of neurons? The weight of the brain? Its brain-to-body ratio? Or its volume? Humans do not have the largest brain size in any of the aforementioned senses. The human brain has about 90 billion neurons, weighs *ca.* 1.1 to 1.4 kg, and has a volume of 1,300 cm^3^. However, the brain of an elephant has three times the number of neurons we have, and the weight and volume of the brain of a sperm whale are six times as much. Meanwhile, ants have a six times larger brain-to-body-mass ratio. A bit of an extreme example showing how cognitive skills and brain size are decoupled is the case of mouse lemurs, whose brains are 1/200th the size of monkeys’ but that perform equally well on a primate intelligence test (Fichtel et al., 2020) Therefore, brain size alone does not make for a more developed mind, either while brain size does not scale with memory information content (Forsdyke, 2014; Forsdyke, 2015). Then what does?

It is plausible to assume that a certain degree of complexity is a mandatory factor for a brain or whatever material structure to display a form of intelligence and cognitive skills. One could think of a measure of ‘brain connectivity’–that is, the number of wirings between neurons (through their axons, dendrites, and synapses) and the speed at which they transmit and receive signals–as an indicator of its complexity and see if it somehow scales with the cognitive functionality. However, MRI studies reveal that all mammals, including humans, share equal brain overall connectivity (Assaf et al., 2020). The efficiency of information transfer through the neural network in a human is comparable to that of a mouse. It is independent of the structure or size of the brain and does not vary from species to species. So, things cannot be as easy as that.

However, what the above-mentioned clinical cases have in common is the presence of the cerebral cortex. In fact, some neurologists or cognitive scientists conjecture that phenomenal consciousness resides in the cerebral cortex. This belief is not unproblematic either.

First of all, because the neocortex exists only in humans and other mammals, one must conclude that birds, fish, octopuses, amphibians, and reptiles are, per definition, all ‘unconscious’ and incapable of having some more or less elementary form of conscious subjective experience. There is no sentience; they do not feel pain, fear, or pleasure or have whatever feeling. They are considered Cartesian automatons or philosophical zombies.

But evidence is beginning to emerge that, for example, the neural correlate patterns of sensory perception in a corvid bird aren’t substantially different from the neural correlate patterns in humans having a similar sensory conscious subjective experience (Nieder et al., 2020). Moreover, one wonders how some birds can also perform amazing cognitive feats despite their forebrains consisting of lumps of gray cells. It turns out that cortex-like circuits in avian birds exist that are reminiscent of mammalian forebrains, and the idea that advanced cognitive skills are possible only because of the evolution of the highly complex cerebral cortex in mammals is becoming less plausible (Stacho et al., 2020). Sufficiently strong evidence concludes that both cephalopods and crustaceans are sentient (Cox et al., 2021). This is unsurprising: Common sense does not really need any scientific proof to accept that ravens, crows, octopuses, or lobsters are sentient beings.

All these findings require an explanation from the physicalist viewpoint, which identifies the mind and consciousness with the brain.

Of course, one could resort to the usual conjecture that neural plasticity explains all things. Neural plasticity certainly plays a role and undoubtedly has its explanatory power. However, in most cases, it remains conjectural and is invoked to fill the gaps that save the paradigm. Some caution would be appropriate. For example, a recent study challenges the idea of adaptive circuit plasticity, according to which the brain recruits existing neurons to take over for those that are lost from stroke. Definitive evidence for functional remapping after stroke remains lacking. Undamaged neurons do not change their function after a stroke to compensate for damaged ones, as the conventional re-mapping hypothesis believed (Zeiger et al., 2021).

Moreover, it is observed that when a brain injury occurs, causing some form of amnesia, what was thought to be lost forever may reemerge into awareness, sometimes after years. Those whose loved ones suffered from dementia may have noted how memory and clarity of thought suddenly and quite surprisingly reappeared in a brief moment of lucidity, called ‘paradoxical lucidity,’ or even ‘terminal lucidity.’ Sometimes, bursts of mental clarity occur shortly before people die. Credible reports document cases in which people with dementia, advanced Alzheimer’s, schizophrenia, or even severe brain damage suddenly return briefly to a normal cognitive state [for a review, see (Nahm et al., 2012); for some more recent findings, see (Batthyány and Greyson, 2021)]. It is hard to recast these brief episodes of lucidity, which last less than 1 h or even a few minutes, by resorting to brain plasticity.

One might also question if, besides the spatial distribution or localization of the neural correlates of consciousness, the intensity of its metabolic activity plays a role in generating a conscious experience. For example, it is well known how the practice of meditation or psychedelic drugs can change our brain chemistry and give rise to the dissolution of the sense of boundaries and intense subjective experiences, respectively. From the perspective of the material monist, which equates mind and brain as being one and the same thing, one assumes that the intensity of ‘mind-expanding’ psychedelics must be directly proportional to an increase in neural activity and connectivity. A dead brain is the cessation of any cerebral activity, in which case we assume there is no consciousness left, while an intensely subjective experience presumably involves high neural activity. One would, therefore, expect to find that the subjectively felt intensity of a hallucinogen proportionally correlates with neuronal activity.

However, the contrary turned out to be the case. A BOLD-fMRI study reported a significant decrease in brain activity–that is, decreased blood flow and venous oxygenation as being inversely proportional to the intensity of the subjective experience reported by the test subjects (Carhart-Harris et al., 2012). The authors of this research remark how this fact is reminiscent of Aldous Huxley’s ‘reducing valve’ metaphor in the brain that acts to limit our perceptions in an ordinary state of consciousness [see also Koch’s take on this (Koch, 2012)]. These findings were later confirmed by further studies with other hallucinogenic drugs such as LSD and ayahuasca (Palhano-Fontes et al., 2015; Carhart-Harris et al., 2016; Lewis, 2017). For a more detailed analysis of this rationale see (Kastrup, 2016). Kastrup also notes how several brain function impairments are accompanied by richer and more intense subjective experiences of self-transcendence (e.g., near-death-experiences associated with dramatically reduced brain function; Kastrup, 2017).

Williams and Woollacott point out how the idea of brain processes attenuating or filtering out mental acuity and broader perceptual awareness is consistent with the literature on meditation studies and Indian non-dual philosophy derived from spiritual practices: Reduced brain activity induced by reduced conceptual activity results in increased cognitive clarity, perceptual sensitivity and awareness expansion (Williams and Woollacott, 2021), suggesting that domains of awareness exist that do not depend upon brain functions.

Furthermore, a neurophenomenological study in the meditating brain showed that the reduction of beta band activity is related to a decreased ‘sense-of-boundaries’–that is, to self-dissolution states giving rise to non-dual awareness (Dor-Ziderman et al., 2016). Similarly, Katyal and Goldin found that deeper meditation experiences are accompanied by increased alpha oscillations (closely linked to inhibitory processing and are often related to the suppression of distractors during attentional cognitive processing) and suppressed theta oscillations (potentially indicating reduced self-monitoring) (Katyal and Goldin, 2021).

Long-time meditators report a state of ‘minimal phenomenal content’, or as a ‘non-dual awareness’ of ‘pure consciousness’, and that could be posited as ‘consciousness as such.’ Investigations on Buddhist meditation suggest distinct correlates of nondual states exist but describe it as ‘non-representational’ awareness (Josipovic, 2014; Josipovic and Miskovic, 2020). Metzinger, instead, conjectures that it could be related to some neurological representational model realized in some brain region with some specific physical properties or neural signatures and correlates that have yet to be discovered Metzinger, 2020. While Katyal argues that the phenomenology of nondual meditative states suggests that a purely non-representational conscious state–that is, a ‘transcendental’ state beyond conscious experience– may transcend any such neural signatures altogether (Katyal, 2022).

### 2.3. The search for the neural correlates of memory

There remain other aspects to explain but that escape a materialistic paradigm with a strikingly similar pattern to that of consciousness and mentation: the neural correlates of memory. Also, in this case, one thing is certain: Memory is not stored in a specific brain area like it is on a digital computer. More than a century of research into the biological foundation of memory has not led to tangible results providing convincing evidence that such substratum exists. This is not a new issue. It dates back to Henri Bergson’s opposition to a reductionist understanding of memory (Bergson, 1896/1912). Bergson considered memory to be of an immaterial and spiritual nature rather than being stored in the brain.

One might assume that information content should somehow scale with brain size. This is not observed, however (Forsdyke, 2014), (Forsdyke, 2016). For example, hemispherectomy in children does not lead to memory impairment (Tavares et al., 2020). How can it be that someone without half of the brain has no measurable memory impairment? We could explain this by resorting to the plasticity of the brain or the functions of residual brain tissues. Or, we could conjecture that memory is stored in both hemispheres; therefore, if one hemisphere is lost, the other remains unimpaired (a hypothesis that could also fit well with supposed evolutionary advantages). Or because it is the diseased hemisphere that is removed in all these cases, Nature might have provided a mechanism that transfers the memories to the healthy hemisphere before surgery. However, we should be aware that these are conjectures, hypotheses, and speculations, not scientifically established truths. Memory storage and retrieval in biological brains remains a largely unexplained mechanism, and no conclusive evidence exists that proves it to be of a physical nature.

Other research that might suggest how and where memories are stored in brains comes from experiments performed on freshwater flatworms called planaria. These creatures can be trained to associate an electric shock with a flash of light. Therefore, one might expect that they must have encoded the experience in their brains.

Flatworm planarians have an incredible self-regeneration ability (Ivankovic et al., 2019). If this worm is cut in half, each amputated body part regenerates as two new fully formed flatworms. Not only does the part with the head form a new tail but the remaining tail also forms a new head with a brain and eyes. In 1959, James V. McConnell showed that the newly-formed planaria with a new brain also maintained its conditioned behavior (McConnell, 1959). The newly-formed living being never received the electric shock and light flash of the training phase and yet it reacted as if it had a memory of the training it had never received.

Memories, if physical, may be stored not only in the brain but also throughout the body, in non-neuronal tissue.

McConnel’s idea was that RNA molecules could transfer memory from one planarian to another as a “memory molecule.” Motivated by this idea, he injected worms with RNA taken from those trained and reported that the training had been transferred. However, further research could not convincingly reproduce McConnel’s experiments.

In 2013, Shomrat and Levin vindicated McConnel’s first experiments by using computerized training of planarians, replacing manual procedures that caused previous test attempts to fail (Shomrat and Levin, 2013). Then, in 2018, Bédécarrats showed how the extracted RNA from a long-term trained sea slug, the aplysia, can induce sensitization in an untrained aplysia (Bédécarrats et al., 2018). This is taken as evidence for the molecular basis of memory and the hypothesis that RNA-induced epigenetic changes lead to the protein synthesis required to consolidate or inhibit memory. These local translations into synaptic proteins determining the neural structure of memory are actually the mainstream engram model.

However, the problem with this hypothesis is that the fastest protein synthesis causes cellular changes in timescales of minutes. How could it possibly be responsible for our ability to store and recall memories almost instantaneously?

Moreover, the still common idea that long-time memory is mapped as synaptic connectivity is challenged by the fact that it is possible to erase synaptic connections while maintaining the same conditioned behavior in the aplysia. Long-term memory and synaptic changes can, at least in some cases, be dissociated (Chen et al., 2014). It has also been shown that the brain tissue turns over at a rate of 3–4% per day, which implies a complete renewal of the brain tissue proteins within 4–5 weeks (Smeets et al., 2018). If the synaptic trace theory is correct, and since synapses are made of proteins, how can, in the presence of this turnover, long-time memory consolidation be achieved in synaptic strengths and neural connection patterns? Notice how the fact that proteins have short lifetimes is in line with the volatility of synaptic connections. How can considerably volatile changes in synaptic connections underlie the storage of information for long periods (even in the absence of learning; Trettenbrein, 2016; Mongillo, 2017)? If memory is physical, other physical repositories must be viable (DNA, cellular organelles, etc.), or a paradigm shift is necessary.

The search for engrams–that is, the group of neurons supposedly responsible for the physical representation of memory–resorts mostly to the correlation between the memory evaluation based on fear conditioning behavioral tasks of rodents and its presumed associated neural changes. For example, in a series of articles the group of Tonegawa claims to have discovered engram cells (Liu et al., 2012; Redondo et al., 2014; Ryan et al., 2015; Roy et al., 2016). They show how light-induced optogenetic reactivation of mice hippocampal neurons that were previously tagged during fear conditioning, induces a freezing behavior characteristic of fear memory recall. While the same activation of cells in non-fear-conditioned mice, or fear-conditioned mice in another context, did not elicit the same freezing behavior. Therefore, the activation of these context-specific neurons seems to suggest that they act like memory engrams of the specific fearful experience.

However, unclear is what really motivates the freezing behavior. The question is whether the cells’ activation led to the memory retrieval of the fearful experience leading to the freezing behavior, if it activates the fear-like emotional state first before any memory retrieval, or if the mice might stop simply because they perceive an unexpected stimulus that might not be related with any fear or remembrance. Only the first case could potentially support the engram hypothesis, but lacking a first-person account, we will never know. While, on the contrary, the second case would only show that the activation of those cells triggers an emotional state that precedes the memory retrieval, and thus, the activated cells would not represent memory engrams (after all, we know that in humans also, stimulation of specific brain centers can lead to panic attacks associated with traumatic events, but these are not necessarily considered as the physical repository of the trauma memory.) While the third case questions whether mice freezing behavior correlates with fear perception in the first place. A lack of motion could be due to many things, not just fear. Moreover, besides the hippocampus, it is possible to induce freezing by activating a variety of brain areas and projections, such as the lateral, basal and central amygdala, periaqueductal gray, motor and primary sensory cortices, prefrontal projections, and retrosplenial cortex (Denny et al., 2017). It is not clear what the freezing behavior is really about.

This, again, shows how the correlation-causation fallacy based on a loss-of-function lesion rationale should be seen with a more critical eye.

Meanwhile, we are also allowed to speculate about a third complementary alternative. Memories associated to physical cues and lower cognitive processes and computational tasks for deductive, inferential, syntactic, predictive optimization problem-solving are material–that is, implemented in a synaptic and molecular basis for consolidation of learned behavior, fact learning, pattern recognition, recording and retrieval of representational content, external sensory cues and other physical information [e.g., see (Gershman, 2023), and that is also an interesting account of the puzzle of the biological basis of memory]. While other memories may be associated to higher cognitive functions involving inductive, non-algorithmic tasks and conceptualizations–that is, memory consolidation and recall of abstract thoughts, semantic categories, and non-representational forms of introspective intuitive cognition and creative expressions that may go beyond a Turing-machine-like information processing [e.g., see (Marshall, 2021), or, for alternatives such as ‘extracorporeal information storage’, see also (Forsdyke, 2015)].

### 2.4. Cognition without a brain

As a concluding note, it is worthy of mention that an increasing body of evidence shows that an at-least elementary form of cognition is already present and working in multicellular and single-celled lifeforms, without any neural substrate. Research in plant biology demonstrates how vegetal and cellular life shows elements of cognitive behavior that were not suspected or were simply considered impossible without a brain. There is extensive literature now that, especially in the last decade, has consistently shown how plants change behavior and adapt, respond predictively, possess some form of memory, resort to air and underground communication systems based on chemical, visual, and acoustic signals, have learning abilities and can evaluate their surroundings, make decisions, and have a cooperative behavior. It is not inappropriate to speak openly of a ‘minimal’ or ‘proto-cognition’ of cells, what is now called ‘basal cognition’. For some reviews see Trewavas (2017), Gershman et al. (2021), and Lyon (2015).

Some climbing plants exhibit an anticipatory prehensile mechanism and able to purposefully plan its movements by an ‘approach-to-grasp’ behavior *before* having any physical contact with a support (Guerra et al., 2019). Other aspects could be mentioned, such as plants’ adaptive changes that reflect developmental decisions based on ‘root-perception.’ Having no central nervous system or information processing centers, roots are, nonetheless, *“able to integrate complex cues and signals over time and space that allow plants to perform elaborate behaviors analogous, some claim even homologous, to those of intelligent animals,”* as Novoplansky describes it (Novoplansky, 2019).

Several experiments with unicellular creatures have made it clear that conditioned behavior in single cells exists as well and is comparable in its complexity to that of plants.

An example could be the evidence of conditioned behavior in amoebae. It could be shown how the motility pattern of the *Amoeba proteus* under the influence of the two stimuli is consistent with associative conditioned behavior (De la Fuente et al., 2019).

A quite surprising ‘brain-less problem-solving’ was (re-)discovered in another protozoan. In 1906, the American zoologist Herbert Spencer Jennings noted how the Stentor roeselii could escalate actions to avoid an irritant stimulus by a complex hierarchy of avoidance behaviors in which the protozoan first enacts a strategy, sees if it works, and if not, resorts to another strategy in a series of attempts to solve a problem. One hundred and 13 years later, in 2019, Jennings’ observations were confirmed (Dexter et al., 2019).

Another notorious example of non-brain-centered cellular cognition is that of the *Physarum polycephalum*, a large amoeba-like slime mold plasmodium that exhibits several skills and behavioral patterns that could be labeled as ‘proto-intelligent’. For example, it can find the minimum length between two points in a labyrinth, and minimize the network path and complexity between multiple food sources (Nakagaki, 2004). Learning processes of habituation with anticipating conditioned behavior was shown as well (Saigusa et al., 2008). For an in-depth review on slime molds see also (Reid, 2023).

Finally, worth a mention is the behavior of the simplest life form, namely, bacteria. These also can sense the environment, actively move within it, target food, avoid toxic substances, and meaningfully change their swimming direction. Most evident is this behavior when they come together forming a bacterial community that shows surprising problem-solving abilities. Bacteria communicate with each other and coordinate gene expression, which determines the collective behavior of the entire community to achieve a common goal with collaborative problem-solving abilities [for a review of bacteria’s behavior see (Lyon, 2015)].

If and how this basal cognition may also imply instances of phenomenal consciousness–that is, some form of more or less ‘basal sentience’–is debatable but can be substantiated by arguments that aren’t exclusively philosophical (Segundo-Ortin and Calvo, 2021). More recently, Parise et al. reviewed the ecological literature, suggesting the existence of an “extended cognition”–that is, a paradigm where one no longer considers the brain as the exclusive seat of cognition, but generalizes it to environmentally extended cognitive processes (Parise et al., 2023).

## 3. Discussion

The paper presented a series of neurological and biological observations whose implications remain controversial. This overview started by questioning the assumption of a lesion-based sufficiency criterion that identifies the causal relationship between the impairment of a specific cerebral area and the, thereby, assumed suppression of phenomenal consciousness and/or cognitive processes, as proof of a material monistic mind-brain identity interpretation. Motivated by this assumption we asked whether the idea of a specific brain area, structure, or its related activity, as being responsible for the qualitative and subjective experiences is consistent with the evidence, and pointed out the lack of conclusive evidence that the phenomenal dimension and singularity of the sense of self-hood, together with its higher cognitive functions is disrupted despite large impairments, suggesting that the hypothesis of a (local or global) brain-based ‘seat of consciousness’, if not inconsistent, must be too simplistic.

Some other neurological aspects of the mind/consciousness-brain relationship were investigated, such as the non-trivial scaling between cerebral size and neural complexity with intelligence, the hypothesis of the cerebral cortex as a center for subjective experience, by comparing it in humans and in other non-mammals, and we examined if and how far neural plasticity alone can be invoked to explain the recovery of cognitive functionalities. Of particular interest is the fact that, contrary to expectations, an inverse relationship between brain activity and conscious experience exists. Reduced brain activity leads to increased cognitive clarity and awareness expansion, seemingly suggesting that at least some aspects of our conscious experience do not depend upon the intensity of brain activity.

The now more than a century longstanding search for the physical basis of memory and memory engram cells was examined. While the predominant paradigm favors the engram hypothesis, here we highlighted how several findings challenge the conventional materialistic view. Observations like memory retention in hemispherectomy cases and planaria’s regenerative memory, along with the limitations of protein synthesis as an explanation and volatility of synaptic connections raise doubts about synaptic trace theory.

Finally, emerging evidence in plant and cellular biology challenges the assumption that all cognition requires a neural substrate. Plant and cellular lifeforms exhibit forms of basal cognition, with abilities including adaptation, memory, communication, learning, decision-making, and problem-solving. Notable instances include the slime mold intelligent behaviors (Reid, 2023) and bacterial communities’ coordinated problem-solving abilities, demonstrating that cognition is not exclusive to organisms with brains (Dinet et al., 2021).

Overall, these findings do not support the mind-brain identity ontology so straightforwardly as is commonly believed. The much too often unquestioned assumption that sees the nervous system as a sine-qua-non condition for conscious experience and cognitive behavior is challenged and we are equally allowed to consider cognition and sentience, not as emerging epiphenomena but as inherent ‘pre-neuronal’ aspects of life.

Of course, ‘pre-neuronal’ does not necessarily mean ‘pre-physical.’ These findings do not refute physicalism in and of themselves. Each of the cited neurobiological facts, when considered separately, may still be saved by several speculations inside the limitations dictated by material monism. The left column of the following table summarizes the findings discussed. The right column furnishes the possible interpretations that could, in principle, save a material monistic paradigm.

**Table 1:** 

Apparent lack of mind-brain identity correlations	Possible interpretations that could save the mind-brain identity theory
Corpus callosotomy and hemispherectomy keep selfhood unified.	Residual subcortical structures may connect the two hemispheres preventing ‘self-splitting’.
Cerebellar agenesis leads to only mild or moderate motor deficits.	Neuroplasticity: The remaining hemisphere takes over the tasks of the missing one.
Hydrocephalus can be quite extreme without necessarily leading to mental impairment.	Neuroplasticity again: Brain tissue may not be lost but only compressed maintaining its functionality.
The hypothesis of the cerebral cortex being the ‘generator’ of conscious experience is contradicted by research on congenitally decorticated children and non-mammalians.	What do we know about what it is like to be a bird?
Thalamus stimulation acts as a ‘gate of consciousness’, not as its ‘generator.’	The thalamus is a hub that ‘modulates’ consciousness; it does not ‘generate’ consciousness.
Brain size (nr. of neurons, mass, volume) does not correlate with cognitive skills.	A minimal nr. of neurons is necessary, then size does not necessarily scale with intelligence.
The brain’s complexity (connectivity, efficiency of information transfer) does not correlate with cognitive skills.	Complexity is more than connectivity and information transfer.
Evidence for engram cells remains debatable, and no memory loss was observed in hydrocephalus or hemispherectomy.	Progress has been made, it is only a matter of time before we will discover the physical basis for memory.
The intensity of psychedelic-altered states of consciousness inversely scale with network disruption.	Maybe psychedelic experiences are unfolding in the brain all the time in the form of unconscious processes. Psychedelics may present it to the surface awareness.
Basal cognition exists without a brain, like in plants and cells.	Will sooner or later be explained away by complicated cell signaling adaptive processes.

However, taken together the lack of these correlations, if we see things jointly in a wider context, that is, without selectively limiting our attention to the single phenomenon seen in isolation, and by taking a coherent integral view in which each phenomenon is seen collectively as the expression of a deeper causal principle underlying the entire pattern, another ontology that does not need such a plurality of physical interpretations is possible. A non-physicalist standpoint that sees mind and consciousness not as an epiphenomenon of matter but, rather, fundamental primitives that manifest *through* the material substrate (e.g., by what James called a ‘transmissive’ rather than ‘generating’ function) in line with a dualistic, idealistic, or other post-material worldviews. A viewpoint, that does not assume a mind-brain identity as a given apriorism but rather sees consciousness and mind as fundamental, with the brain a ‘physical mind’ that mediates information from and to a non-physical mind, could accommodate the above-listed lack of correlation between neurological and experiential/cognitive phenomenality inside a paradigm that does not need all these mechanistic conjectures.

Anyway, a future direction of systematic research that does not always assume the mind-brain identity as a given fact and leaves doors open to other perspectives, would be sufficient to potentially lead to powerful new insights that were previously overlooked. A possible future generalist approach, that does not necessarily impose one or another metaphysical worldview but starts with the assumption of a ‘post-material psychology’, could be a line of research (Beauregard et al., 2018). The mind–body problem and the hard problem of consciousness remain controversial issues more than ever, but non-physical ontologies of mind and consciousness are far from having been expunged by science. We have the right to explore these as a viable option not despite but, to the contrary, because of neuroscientific evidence that has been selectively dismissed for too long but cannot be ignored forever–if we can connect the dots.

## Data availability statement

The original contributions presented in the study are included in the article/supplementary material, further inquiries can be directed to the corresponding author.

## Author contributions

The author confirms being the sole contributor of this work and has approved it for publication.
